# Mr. Hume, on the Cupping-Glass in Hydrophobia

**Published:** 1804-10-01

**Authors:** J. Hume

**Affiliations:** Long Acre


### *To the Editors of the Medical and Physical Journal.*

Gentlemen,

On reading in the Medical and Physical Journal for April, the ingenious method, proposed by Mr. Hardman, to open an abscess by means of an exhausted cupping-glass, it naturally occurred to me, that the same mode could be most effectually employed in various other instances, where it may be requisite to extract morbid or extraneous fluids, especially when no time should be lost to afford relief.

Those who are conversant in Surgery, and its daily practice, may, possibly, suggest a great variety of other cases, in which this mode of operating can be advantageously followed; there is, however, one, of the utmost importance, in which it may afford an immediate cure, and completely prevent the most afflicting and direful malady, to which a living body is liable, that is, *Hydrophobia.*

It can only be ascertained by fair and repeated experiments, whether we can depend upon this as a secure preventive; and it certainly should not be condemned or refused until we have had the most undeniable proofs of its inefficacy.

The moment an accident of this kind has happened is, evidently, the only period to apply the glasses, with any prospect of success. A more powerful degree of suction can easily be obtained by the use of a glass receiver, furnished with a stop cock, and exhausted by the air-pump. The mouth of this vessel should not be very narrow, as it may be proper and convenient to cover all the tooth-marks at once, if possible, or in as rapid succession as may be. The blood, if it flow, should be instantly wiped off with a sponge; and it would be prudent to repeat the operation at least once more.

Besides canine-madness, this method should be tried in all all similar cases; and the very same means pursued, whether the wound has been inflicted by a rabid or poisonous animal, or by a poisoned instrument.

I may, perhaps, have stumbled upon what has already been publicly noticed and recommended by others; if so, I can only assure you, this communication arises from the best intention, and, should it be found destitute of original merit, I hope you will reject it. I am, &c.

*Long Acre, Sept.* 13, 1804. J. HUME.

